# Oncology nurses‘ beliefs and attitudes towards the double-check of chemotherapy medications: a cross-sectional survey study

**DOI:** 10.1186/s12913-018-2937-9

**Published:** 2018-02-17

**Authors:** D. L. B. Schwappach, Katja Taxis, Yvonne Pfeiffer

**Affiliations:** 1 Swiss Patient Safety Foundation, Asylstr. 77, 8032 Zurich, Switzerland; 2 Institute of Social and Preventive Medicine (ISPM), University of Bern, Bern, Switzerland; 3 Department of Pharmacy, Unit of Pharmacotherapy and Pharmaceutical Care, University of Groningen, Groningen, The Netherlands

**Keywords:** Patient safety, Medication errors, Oncology, Double-check, Survey

## Abstract

**Background:**

Double-checking medications is a widely used strategy to enhance safe medication administration in oncology, but there is little evidence to support its effectiveness. The proliferated use of double-checking may be explained by positive attitudes towards checking among nurses. This study investigated oncology nurses’ beliefs towards double-checking medication, its relation to beliefs about safety and the influence of nurses’ level of experience and proximity to clinical care.

**Methods:**

This was a survey of all oncology nurses in three Swiss hospitals. The questionnaire contained 41 items on 6 domains. Responses were recorded using a 7-point Likert scale. Multiple regression analysis was used to identify factors linked to strong beliefs in the effectiveness of double-checking.

**Results:**

Overall, 274 (70%) out of 389 nurses responded (91% female, mean age 37 (standard deviation = 10)). Nurses reported very strong beliefs in the effectiveness and utility of double-checking. They were also confident about their own performance in double-checking. Nurses widely believed that double checking produced safety (e.g., 86% believed errors of individuals could be intercepted with double-checks). In contrast, some limitations of double-checking were also recognized, e.g., 33% of nurses reported that double checking caused frequent interruptions and 28% reported that double-checking was done superficially in their unit. Regression analysis revealed that beliefs in effectiveness of double-checking were mainly associated with beliefs in safety production (*p* < 0.001). Nurses with experience in barcode scanning held less strong beliefs in effectiveness of double-checking (*p* = 0.006). In contrast to our expectations, there were no differences in beliefs between any professional sub-groups.

**Conclusion:**

The widespread and strong believe in the effectiveness of double-checking is linked to beliefs about safety production and co-exists with acknowledgement of the major disadvantages of double-checking by humans. These results are important factors to consider when any existing procedures are adapted or new checking procedures are implemented.

**Electronic supplementary material:**

The online version of this article (10.1186/s12913-018-2937-9) contains supplementary material, which is available to authorized users.

## Background

The double-check of medications prior to administration is a widely implemented safety procedure aiming at intercepting errors at “the very last mile” of patient care in hospitals and other institutions. Despite its wide diffusion in healthcare, there is only very limited evidence supporting double-checking as an effective measure for error detection: A recent systematic review concluded that only little research has been done into the effectiveness of double-checking and that its evidence is insufficient [1]. A classic study compared double- vs. single-checking of medications and reported a lower error rate with double-checking (2.12 vs. 2.98 per 1000 medications administered). However, the authors questioned the clinical relevance of this small effect [2]. The Institute for Safe Medication Practices (ISMP) places “checklists and double check systems” on a medium rank in a rank order of error reduction strategies (rank 4 of 7 most effective strategies). In a recent “Medication Safety Alert” the ISMP recommends the implementation of the double-check, but judiciously, and in a standardized process [3].

Research has also brought up several issues which question double-checking as a safety enhancing policy: double-checking increases the workload of nurses; it needs full attention doing the task which makes it vulnerable to distractions (environmental influences such as interruptions or noise) [4]; and it diffuses responsibility [5]. Furthermore, as two nurses are needed for a double check, the checks also significantly increase the number of interruptions during a work day, which increases the chance for errors in medication administration and verification [6]. Given the challenges of systematic implementation of high-evidence interventions in many clinical areas [7], it is surprising that a safety-oriented strategy which requires considerable resources for the individual (in terms of cognitive load) and for the organization (in terms of personnel and time), is diffused and proliferated despite a major lack of evidence. We therefore hypothesized that this may be explained by strong subjective beliefs among healthcare professionals (or certain groups of them) that double-checking has considerable, positive effects on safety. Such positive attitudes may foster implementation of the double-check, irrespective of scientific validation. The evidence on this issue is mixed and ambiguous [1, 4, 5, 8–10]. Although double-checking is often recommended as effective “best practice” in detecting and preventing drug administration errors, health professionals perceive the process as inconsistent [5, 10]. O’Connell et al. report a study in which the double-check procedure was de-implemented [11]: Nurses’ held strong views against single checking before the change in practice. However, 1.5 years after the implementation of the single-check, results of a second survey indicated that nurses welcomed the single check medication procedure. They felt more confident using single checking and perceived that it made them more accountable for administering medications.

From research on subjective theories, beliefs and experiences how errors occur and how safety is produced in safety critical organizations [12], we think that clinicians’ views about the effectiveness of double-checking procedures are connected to such individual subjective theories: Basically, human double-checking is based on the belief that errors can be minimized by other individual’s compensatory behaviour [12]. The expectation that redundant human checks will increase safety involves individual-focused beliefs that errors are made by unaware individuals and safety can be produced by “duplicating human awareness”. These beliefs disregard the fact that both individuals are exposed to the same root causes and influences contributing to potential errors (i.e., environmental factors such as noise; heuristics and biases during cognitive tasks) [13]. Furthermore, simply knowing that a co-worker will verify a particular medication for a patient may negatively affect motivation and result in the tendency to expend less effort (i.e., “social loafing” [14]). According to Schöbel and Manzey [12] this can result in greater diffusion of responsibility and thus decreased system safety. Indeed, this is backed-upped by nurses’ reports that that the double-check would reduce the perceived responsibility of individuals because others would pick up potential mistakes [4, 5].

In oncology, a large fraction of medication errors occur during the administration stage [15]. Thus, in theory, double-checking before administration could be a very valuable process for intercepting these errors. As many medications in cancer care involve high-risk drugs with a potential to cause severe harm in a very vulnerable population, even safety measures with limited effectiveness may be justified if alternatives are lacking. In particular, double-checking may serve as an important social and psychological support mechanism by staff administering these high-risk drugs on a daily basis irrespective of its functionality in detecting errors. Even though checking, verifying and administering medications is a central task in cancer care, data about oncology nurses’ attitudes towards, beliefs in and perceptions of double-checking is completely lacking. This study addresses this gap and examines oncology nurses’ beliefs about the double-check.

The primary aim of the study was to assess perceived subjective norms towards the double-check, self-efficacy in performing checks, beliefs in the effectiveness of the double-check, beliefs in safety production, and perceived limitations of the double-check in clinical practice. The secondary aim was to investigate the relationship between subjective beliefs in error and safety production, nurses’ experience, proximity to clinical care and their beliefs in the effectiveness of the double-check. Westbrook et al. identified that nurses’ level of experience has an important influence on the likelihood of committing a medication error. Therefore, the expectation that level of experience would also be associated with beliefs about the double-check seemed justified to us [16]. We expected that intense practice of medication verification procedures “at the sharp end” would influence beliefs about how errors evolve, and amplify experiences with flaws of redundant checks performed by humans, and thus affect the perceived effectiveness of the double-check in improving safety negatively.

We thus hypothesised that differences in attitudes and beliefs would exist according to nurses’ level of experience. Second, we had the hypothesis that perceived effectiveness of the double-check is associated with nurses’ beliefs in safety production, their perceived subjective norms, self-efficacy in performing the checks and experienced limitations of the double-check.

## Methods

### Survey development

The survey was developed by the investigators based on a review of the literature and consultations with experts and clinical staff. Site visits, field observations and informal talks with clinicians were conducted to gain an understanding of how the double-check is implemented in routine clinical care at the participating units. The survey consisted of two main sections: The first section (not reported herein) assessed practice patterns and experiences with double-check procedures. Using scenario-type questions, the variability of checks implemented at different units, for different types of medications, and the practice and results in conducting these checks were assessed in detail [17]. The second survey section (reported herein) assessed attitudes and beliefs in double-checking medications. During survey development items were generated, adapted, or rejected in an iterative procedure by the research team. In a first step, an extensive list of items was generated focusing on the constructs to be measured and with the field observations in mind. These constructs were:

‘Beliefs in benefits and effectiveness of the double-check’ represents the perceived value of double-checking and its role in ensuring medication safety. ‘Beliefs in own performance / self-efficacy’ aims to measure nurses’ confidence in their abilities to conduct double-checking under the constraints of their typical environmental working conditions and to achieve the intended results. ‘Perceived subjective norms and coherence’ address the shared and collectively of values and expectations linked to double-checking. ‘Beliefs in safety production’ represents mental models of how and why the double-check generates safety and the contribution of humans to error and error detection. ‘Limitations of the double-check in clinical routine’ covers factual and potential experiences and ideas of the deficits of double-checking.

In two iterative rounds, items were evaluated by researchers individually and then discussed. Items were dropped or re-worded with the aim to achieve a concise set of items mapping the target constructs. Main reasons for rejection and adaption were: item complexity, poor wording, similarity, ambiguous mapping to constructs [18]. The team had different professional backgrounds (psychology, pharmacy, health services research, and nursing). Three were patient safety researchers experienced in survey design. The survey is available as online supplement (Additional file 1).

Finally, 41 items were included: 7 items assessed beliefs in effectiveness and utility of double-check; 9 items assessed beliefs about safety production; 10 items assessed limitations of the double-check in routine clinical care; 6 items were related to perceived subjective norms and coherence of double-check attitudes in the unit; and 9 items assessed beliefs in own performance and self-efficacy. To avoid ordering effects (i.e., intercorrelations of items due to placement of questions), items were not presented as scales but as two blocks. The first block included all items except those assessing beliefs in own performance and self-efficacy under the heading “your thoughts about double-checking”. The second block used the heading “about your individual situation” and included the remaining items. Within blocks, items were randomly ordered. Tables 2, 3, 4 and 5 present the final item wordings. A 7-point Likert response scale was used for all items, with the anchors labelled “completely agree” and “completely disagree”. At the end of the survey, respondents were asked to complete socio-demographic and work-related items. After selecting and wording the items, six experts from nursing, oncology, clinical pharmacy, and hospital risk management gave feedback on a draft.

The survey was then pretested for clarity and wording in a sample of *n* = 39 nurses from two hospitals not participating in the main study. Pre-testers were asked to complete the survey and provide written feedback on comprehensibility, difficulties in responding, clarity of wording, and further comments. Pre-test results were analysed in terms of missing values, variance, floor and ceiling effects, and unevaluable responses [19]. Only minor adjustments were made to item wording. For example, we changed the term “co-worker” to “co-working nurse” because few responders asked whether “co-worker” would include doctors. As part of instrument assessment and to verify the constructs as scales for regression analysis, we conducted exploratory factor analysis for each scale (Kaiser-Criterion, factor extracted if Eigenvalue > 1, oblique promax rotation). This analysis confirmed that all items entering the outcome scale (“beliefs in effectiveness of the double-check”) loaded on one factor (Eigenvalue = 2.3) andall other factors had very low Eigenvalues (< 0.25). Similar results were obtained for the other scales. Cronbach’s Alpha ranged between 0.64–0.75 for the subscales.

### Sample

Three Swiss hospitals participated with their oncology departments (two university hospitals, one large regional hospital). Each hospital took part with oncology wards and ambulatory units. All sites have different double-checking policies in place. At all sites, double-checking was required for all antineoplastic drugs whereas there existed differences on double-checking requirements for other medications, e.g., premedications. Double-checking was common practice for several years at least for some medications at all units. All qualified nurses working on the participating units received the paper survey together with a pre-paid envelope and a chocolate bar via internal mail. They had two weeks time to return the survey. Return of the survey was regarded informed consent. The study was deemed exempt by the Cantonal ethics committee (KEK ZH Nr. 34–2015).

### Analysis

Responses were analysed using descriptive and inferential methods. Cases with missing data were deleted pairwise in analyses. Item nonresponse was low and ranged between 1 and 4% per item. Negatively worded survey items were recoded (indicated by a “*” in the tables) to allow calculation of meaningful scale scores. In addition, survey items were dichotomized to “agreement” (scores 5 to 7 on the Likert scale) and “neutral/disagreement” (scores 1 to 4) for easier interpretation. Mean scale scores were computed by averaging responses over the set of items assessing a construct (e.g., mean of items of “beliefs in effectiveness”). To assess our hypothesis that clinical experiences are associated with beliefs about the double-check, we examined differences in mean scale scores between a) responders from wards and ambulatory infusion units, b) nurses with more vs. less than 5 years clinical expertise in oncology, c) nurses with more vs. less than 40 h per week in direct patient care, and d) nurses with vs. without expert or managerial qualifications using ANOVA. Multiple regression analysis was conducted to determine which factors influencing strong beliefs in the effectiveness of the double-check (dependent variable). Beliefs in safety production, perceived norms, self-efficacy, limitations of the double-check, and personal and work-related characteristics were included as predictors (independent variables). All tests were two-sided and a *p*-value < 0.05 was considered statistically significant.

## Results

Of the 389 distributed surveys, 274 were completed and returned (n_hospital A = 55, n_hospital B = 148, n_hospital C = 71; overall response rate = 70%). Sample details are provided in Table 1. Mean scores and frequency of agreement to a statement are presented in Tables 2, 3, 4 and 5.

**Table 1:** Characteristics of survey responders (*n* = 274)

Characteristic	Responders	
	n	%
Female Gender	240	91
Age, mean (SD) years	37 (10)	
18–25 years	31	12
26–40 years	149	56
41–55 years	67	25
56–65 years	17	6
Qualification^a^		
Qualified nurse	205	76
Oncology nursing expert	42	16
Head nurse	17	6
Other	4	1
Primary place of work		
Ward / Oncology day care unit	220	82
Ambulatory infusion unit	48	18
Weekly hours in direct patient care		
< 10 h / week	14	5
10–25 h / week	60	23
26–40 h / week	123	47
> 40 h / week	67	25
Experience with barcode scanning (e.g. blood products)	123	46
Preparation of cytostatics at unit	82	31
Years of practice in oncology, years		
< 1 year	25	10
1–5 years	89	36
6–10 years	55	22
> 10 years	79	32

^a^Categories may not sum up to 100% due to missing values

**Table 2:** Survey items, means, standard deviations (SD), % agreement: Beliefs in benefits and effectiveness of the double-check

Nr	Item	Mean^a^	SD^a^	% Agree
Beliefs in benefits and effectiveness of the double-check (alpha = 0.75)		5.7	0.8	
F09	Nowadays, double-checking is regarded “good practice” in cancer care.	6.3	0.9	94.8
F11	A hospital needs to implement double-checking for dangerous drugs because otherwise it will look bad in case of error.	5.9	1.4	84.3
F15^a^	Technical solutions like barcode /bedside scanning would be better alternatives to double checks by humans.	4.1	1.6	33.8
F16	The double-check is the central task for medication safety.	5.8	1.3	86.5
F17	The resources needed for the double-check are justified for the additional safety it produces.	6.2	1.1	91.8
F24	I’m convinced about the benefits of the double-check how it is done here.	6.1	1.0	94.4
F27	If the double-check would be deimplemented, threats to patients would increase considerably.	5.7	1.4	79.9

^a^Values are reverse-coded for negatively worded items

**Table 3:** Survey items, means, standard deviations (SD), % agreement: Self-efficacy and perceived norms and coherence

Nr	Item	Mean^a^	SD^a^	% Agree
Beliefs in own performance / self-efficacy (alpha = 0.70)		5.9	0.6	
F28	I know what matters most for a good double-check.	6.6	0.6	99.3
F29	I can give my colleagues critical feedback when they are inattentive at the double-check.	5.9	1.2	90.2
F30	I’m sure that I make a good double-check despite pressure and interruptions.	5.6	1.3	86.8
F31	I know exactly which checks are required for a certain medication.	6.1	1.0	90.9
F32^a^	It can happen that I forget the double-check.	5.5	1.8	18.1
F33	I’m certain that I do the double-check correct.	6.2	0.9	95.9
F34	I realize myself when I’m inattentive and ‘asleep at the wheel’.	6.1	1.0	95.1
F36	I’m confident I detect every important inconsistency in medications.	5.5	1.2	83.0
F37^a^	Sometimes I rely too much on the second person at the double-check.	5.3	1.5	15.2
Perceived subjective norms and coherence (alpha = 0.64)		5.1	1.0	
F39^a^	There are very different views about the importance of double-checking at my unit.	4.5	1.8	29.3
F40^a^	Staff at my unit is rather sceptical about double-checking.	5.5	1.4	8.0
F42^a^	Rules at our unit are somewhat stricter that what we actually do in double-checking during routine care.	5.5	1.5	11.0
F46	High quality of double-checking is very important to nursing leaders of my unit.	5.9	1.3	80.2
F47	High quality of double-checking is very important to physician leaders of my unit.	4.7	1.8	48.0
F50^a^	Some colleagues here rely too much on the second person at the double-check.	4.5	1.8	30.3

^a^Values are reverse-coded for negatively worded items

**Table 4:** Survey items, means, standard deviations (SD), % agreement: Beliefs in safety production

Nr	Item	Mean	SD	% Agree
Beliefs in safety production (alpha = 0.64)		5.3	0.8	
F10	The double-check introduces a final moment of silence and concentration before medications are being administered.	5.9	1.3	87.4
F13	Most medication incidents are attributable to a lack of attention of individuals.	5.2	1.5	69.9
F18	Errors of single individuals can be intercepted with redundant checks by humans.	5.7	1.2	85.8
F19	The primary purpose of the double-check is to prevent errors in medication preparation.	4.8	1.7	61.0
F20	Responsibility is shared with the double-check.	4.3	2.0	49.4
F23	I’m particularly vigilant if a double-check is required for a medication, because it must be a dangerous drug then.	5.1	1.7	66.4
F25	The primary purpose of the double-check is to detect prescribing errors.	4.3	1.6	50.9
F26	Environmental factors such as illumination, interruptions etc. increase the risk for errors considerably.	6.0	1.2	90.3
F35	To me, it feels reassuring to know that dangerous drugs which I prepared will be checked by a co-worker.	6.3	1.0	95.1

**Table 5:** Survey items, means, standard deviations (SD), % agreement: Limitations of the double-check in clinical routine

Nr	Item	Mean	SD	% Agree
Limitations of the double-check in clinical routine (alpha = 0.75)		3.3	0.9	
F14	Most errors happen directly before medication administration, after all double-checks have been conducted.	3.6	1.7	32.1
F21	The double-check gives a false sense of safety.	2.9	1.6	17.7
F22	In everyday practice, the double-check is often superficial routine.	3.2	1.7	27.6
F38	I often feel distracted from my work when I’m called to a double-check by a colleague.	3.5	1.9	33.1
F43	There are times of the day or week where a good double-check is not feasible.	2.5	1.8	17.3
F44	We make too many checks of medications.	2.2	1.4	6.8
F45	The double-check makes our workflow more complicated.	3.0	1.8	25.9
F49	It happens that two persons make the same mistake during a double-check (e.g., calculation error).	4.0	1.9	44.7
F51	Misleading information or the way, orders are filled frequently complicate the double-check.	4.7	1.8	58.1
F52	The different speed of staff (e.g., in reading) makes the double-check difficult.	3.0	1.8	25.0

### Descriptive results

#### Beliefs in effectiveness and utility of double-check

Overall, among nurses in our study strong beliefs in the effectiveness of the double-check procedure were common: The vast majority agreed that the double-check is the central task for medication safety (87%; F16 Table 2) and that the resources needed are justified by the additional safety level achieved (92%; F17 Table 2). Contrary, only 34% agreed that technical solutions like barcode /bedside scanning would be better alternatives to double checks by humans (F15, Table 2). Considerable threats to patient safety if the double-check would be eliminated were expected by 80% (F27, Table 2).

#### Beliefs in own double-check performance / self-efficacy

Responders shared strong, positive beliefs about their own double-check performance: Virtually every participant stated that s/he knows what matters most for a good double-check (F28, Table 3). Also, the vast majority was confident to make the double-check correctly, even under time pressure and when interrupted (F30, Table 3). Nurses also reported to be self-aware about being distracted (F34, Table 3).

#### Perceived subjective norms and coherence of double-check in the unit

There was less consistency in perceived norms and perceived coherence of practice at the units: For example, 29% agreed to the statement that there are very different views about the importance of the double-check at their unit (F39, Table 3). Leadership support for the double-check was perceived as coming from nursing leaders and to a considerable lesser extent from physician leaders (F46 and F47, Table 3).

#### Beliefs in safety production

A majority of nurses believed that medication incidents are attributable to a lack of attention of individual staff (F13, Table 4) and that these errors by individuals can be intercepted by redundant checks (F18, Table 4). These beliefs coexist with unequivocal agreement on the impact of environmental factors on patient safety (F26, Table 4). Nurses felt reassured by knowing that a co-worker will double-check dangerous drugs (F35, Table 4) and half of responders agreed that responsibility is being shared with the double-check (F20, Table 4). However, results were ambiguous about the main target of the double-check: 51% and 61% of responders agreed that the main purpose of the double-check is to detect prescribing and preparation errors respectively (F25 and F19, Table 4).

#### Limitations of the double-check in routine clinical care

Results presented in Table 5 provide insight into perceived common limitations of the double-check in clinical routine. Many nurses feel distracted from work by supporting colleagues with the double-check (F38, Table 5) and perceive workflow to be complicated by double-checking (F45, Table 5). 28% of nurses agree that the double-check is often superficial routine and nearly 50% acknowledge that two co-workers sometimes make the same error during double-check (F22 and F49, Table 5). 18% admit that the double-check gives a false sense of safety (F21, Table 5). Notwithstanding the reported limitations, only 7% of nurses said that the number of checks is too high (F44, Table 5).

#### Differences in double-checking constructs according to level of clinical experiences

In contrast to our hypothesis, there were only few and spurious differences in mean scores in relation to nurses’ level of experience and proximity to clinical care (Table 6):

**Table 6:** Group-level differences in double-checking constructs

		Beliefs in effectiveness	Beliefs in safety production	Perceived norms	Self-efficacy	Limitations of the double-check
Primary place of work	On ward	5.7 [0.8]	5.3 [0.7]	5.0 [1.0]**	5.9 [0.6]	3.3 [0.9]
	Ambulatory infusion unit	5.8 [0.7]	5.2 [0.9]	5.5 [1.1]**	5.9 [0.7]	3.1 [1.2]
Years of practice in oncology	≤ 5 years	5.7 [0.8]	5.3 [0.8]	5.0 [1.0]	5.8 [0.7]	3.2 [1.0]
	> 5 years	5.7 [0.8]	5.3 [0.8]	5.2 [1.0]	5.9 [0.6]	3.3 [1.0]
Weekly hours in direct patient care	≤ 40 h	5.7 [0.8]	5.3 [0.8]	5.1 [1.0]	5.8 [0.6]*	3.3 [1.0]
	> 40 h	5.7 [0.8]	5.3 [0.8]	5.1 [1.0]	6.0 [0.5]*	3.3 [1.1]
Qualification	Nurse	5.8 [0.7]	5.3 [0.8]	5.1 [1.0]	5.9 [0.6]	3.4 [1.0]
	Nursing expert/head nurse	5.6 [1.0]	5.2 [0.8]	5.1 [1.1]	5.9 [0.6]	3.2 [1.0]

Values are mean scale scores [SD]

*Group mean significant different at *p* = 0.0211 after Bonferroni adjustment for multiple testing

**Group mean significant different at *p* = 0.0072 after Bonferroni adjustment for multiple testing

We found strong coherence and no systematic differences in mean effectiveness and safety production beliefs and reported limitations of the double-check between any professional sub-groups. Nurses working more than 40 working hours per week in direct patient care provided slightly higher mean self-efficacy scores compared to those working fewer hours in patient care (6.0 vs. 5.8, *p* = 0.021). Nurses working at ambulatory infusion units provided significantly higher mean scores on the perceived norms and coherence scale compared to nurses working on wards (5.5 vs. 5.0, *p* = 0.007). This difference existed for all items in the scale except for the two leadership support items (F46,F47).

#### Influences on beliefs in effectiveness of the double-check

Beliefs in effectiveness of the double-check were mainly associated with beliefs in safety production (Table 7). Stronger perceptions of the limitations of the double-check in clinical practice were negatively linked to beliefs in its utility. However, this relation was not substantial: A 6-fold increase in mean score in perceived limitations of the double-check in clinical practice corresponds to only a one point decrease in beliefs in double-check effectiveness. Responders with personal experience with barcode scanning (medications or blood products) held less strong beliefs in effectiveness of double-check, even after adjusting for other variables. Self-efficacy, perceived norms and personal and work-related characteristics did not determine beliefs in effectiveness and utility of the double-check.

**Table 7:** Results of regression analysis with beliefs in double-checking effectiveness (mean scale score) as outcome variable

Variable	Coefficient (non-standardized)	95% CI	*P* value
Beliefs in safety production, mean scale score	0.543	0.442,0.643	< 0.001
Perceived norms, mean scale score	0.076	−0.016,0.169	0.107
Self-efficacy, mean scale score	0.065	−0.070,0.200	0.344
Limitations of the double-check, mean scale score	−0.163	−0.255,-0.071	< 0.001
Experienced with barcode scanning	−0.216	−0.368,-0.063	0.006
Nurse (vs. nursing expert/head nurse)	0.059	−0.122,0.240	0.522
Working on ward (vs. ambulatory infusion unit)	−0.119	−0.322,0.084	0.250
Constant	2.884	1.749,4.019	< 0.001
n with complete data	261		
R-sqr	0.43		
Overall model p			< 0.001

## Discussion

To our knowledge, this is the first in-depth assessment of nurses’ beliefs and attitudes towards the double-check of medication, in particular in oncology. We found very strong beliefs in the effectiveness and the utility of the double-check accompanied by a high perceived self-efficacy in performing the double-check and ensuring proper medication verification. This helps to explain the rather unquestioned proliferation of the double-check in practice. Against our expectations, there was a solid consensus in these beliefs among nurses, independent of proximity to clinical care, years of experience, level of training, and whether nurses work at ambulatory infusion units or on wards. Positive attitudes towards the value of the double-check were associated with beliefs about safety production which focus the individual’s attention deficits as source of error. However, some results related to beliefs in safety production also seem contradictory. For example, nurses were ambivalent about which type of errors the double-check primarily addresses (prescription or preparation errors). Prior interview and observational studies [8, 20] have reported non-compliance with unit policies regarding how to do the double-check. The results of our study suggest that non-compliance does not seem to stem from a negative attitude towards checking itself, and not from low perceived subjective norms either, i.e., missing social examples and pressure to execute double-checks [21]. Thus, the causes underlying non-compliance are to be looked for in other factors impeding checking, e.g., time pressure.

However, despite the prevalent and unequivocal positive beliefs in the effectiveness and utility of the double-check, a considerable part of the sample also reported serious limitations of the procedure in clinical practice. Half of responding nurses agreed that it happens that two nurses make the same mistake during double-check, a third of the respondents felt commonly distracted by being called to support a double-check and nearly 30% agreed that the double-check is often superficial routine. Surprisingly, these practical experiences do not seem to develop reluctance towards the double-check or question its effectiveness. Rather both of these evaluations seem to co-exist. Moreover, the majority of the surveyed nurses think that safety would be jeopardized if the checks were not performed and does not consider technical solutions like bedside/barcode scanning a viable alternative to human double checking. The tendency to accentuate the role of humans in safety production has been described as “bad apple theory” [22]. This tendency matches what we identified as a common belief about safety production: we found high agreements with the statements that most medication incidents are attributable to a lack of attention and that human errors can best be caught by other humans performing checks.

Another related contradiction we found in our results is that the respondents are very confident about their own knowledge and double-checking performance, while also a considerable fraction reported that double-checks are often done superficially or that an error was not caught despite the double check. This contradiction could be related to the feelings of responsibility that may be a burden in administering high-risk medication. Remarkably, half of the nurses in our sample agreed with the statement that with the double-check responsibility is being shared and virtually all felt it reassuring to know that a co-worker will check prepared medications. If the burden of responsibility is being shared if a double check is performed, the value of the double-check may also be considered an emotional one. This represents an interesting avenue for future research on the role of double-checking in nurses’ work, specifically in oncology where the drugs administered are highly toxic. It remains unclear whether our results are generalizable to other clinical areas, in particular those with lower utilization of high-risk drugs. Previous research addressed double-checking practices and attitudes in paediatrics and neonatology, also an area with a high frequency of dangerous drug administrations [1, 4, 8, 20]. Future studies are needed to compare double-checking attitudes and beliefs in different clinical areas with different risk profiles of medication errors.

### Limitations

The generalizability of our results is limited by the fact that we sampled only nurses from three hospitals. The response rate was satisfactory for a written survey. As our items and scales were newly developed our results should be regarded preliminary. Further explorations are needed to confirm them as valid operationalizations of the latent constructs we aimed to measure. Still, we think that the results are valuable and innovative in that they link beliefs about the double-check with views on how safety evolves. The high rates of agreement especially with the effectiveness items point to a ceiling effect. We worded the items to include high thresholds (e.g., by including the word “considerably” in item 27) and still observed very strong agreement and positive evaluations of double-checking. Two of the main predictors in the regression analysis (“beliefs in safety production” and “limitation of double-check”) are thematically closer to the outcome variable (“beliefs in effectiveness”) than perceived norms and self-efficacy. This may explain why the other two scales were not significant predictors. In addition, results may be subject to common methods bias as we obtained all results within the same survey. Finally, assessment of subjective theories and beliefs about safety using a self-administered survey method may bring up restricted insights, which are, however, a valuable contribution to the field.

## Conclusions

Oncology nurses strongly believed in the effectiveness and the utility of double checking medication despite reporting limitations of the procedure in clinical practice. As prior literature has identified a lack of evidence regarding the effectiveness of double-checking, and due to the reported disadvantages of double-checking, hospital managements and nurse practitioners may consider changing current practice, for example in designing checking routines more specifically, reduce disturbing environmental influences, or by implementing single checks [5]. In analyzing the attitudes towards double-checking and identifying the strong beliefs in double-checking as a safety enhancing method, this study’s results suggest that a change of checking routines may be difficult to implement in practice. We therefore recommend accompanying a change of checking procedures by training (in accordance with Hewitt et al. [10]) and a prior exchange between nurses and nurse management about their perspectives on the need for checking in the administration process and on how safety is best achieved. As our study showed that the nurses are also aware of limitations of checking, they may be open for such an exchange if they feel taken seriously by management.

In particular, the widespread view that human actions are considered as most effective in safeguarding patient care should be questioned, particularly when trying to implement technical solutions such as barcoded checking. Failures to use such technologies in practice may be due to beliefs and attitudes we identified in our study [23]. Taking on other perspectives such as system design to reduce medication errors (e.g., in reducing distractions during checking by attributing a specific quiet room for double-checking) may over time not only help improving the work processes, but also change the subjective views about double checking, its effectiveness and best way to perform. As those responders experienced with barcode scanning were less convinced about the effectiveness of double-checking, we suggest that the attitudes towards a new checking procedure and its evaluations by the workforce change with the time of use. Future research could shed light on the change of evaluations with the introduction of new procedures and in order to better understand the role and evolution of subjective theories about safety strategies.

## Additional file

Additional file 1: Double-check survey instrument for oncology, translated from German original. (PDF 323 kb)

## Abbreviations

CI: Confidence interval
SD: Standard deviation
