# Supplementary material for: Oncology nurses‘ beliefs and attitudes towards the double-check of chemotherapy medications: a cross-sectional survey study
Source: BMC Health Serv Res. 2018 Feb 17;18:123. doi: 10.1186/s12913-018-2937-9 (PMC5816392; doi:10.1186/s12913-018-2937-9)
Supplement: Supplementary file 1 — Double-check survey instrument for oncology, translated from German original. (PDF 323 kb) [file 12913_2018_2937_MOESM1_ESM.pdf]

On the following pages we will present you five different variants of double-checks as they are conducted in clinical practice. As checking procedures may differ at different hospitals and units they are only broadly characterized.
Please answer the questions with the respect to the presented variant.

Scenario A Verification of order and IV bag:
Nurse A reads out loud data on the order (paper or computer screen). Nurse B verifies against the IV bag and confirms. Nurse B reads out loud data on the IV bag label and Nurse A confirms against the order. One of both administers the medication to the patient.

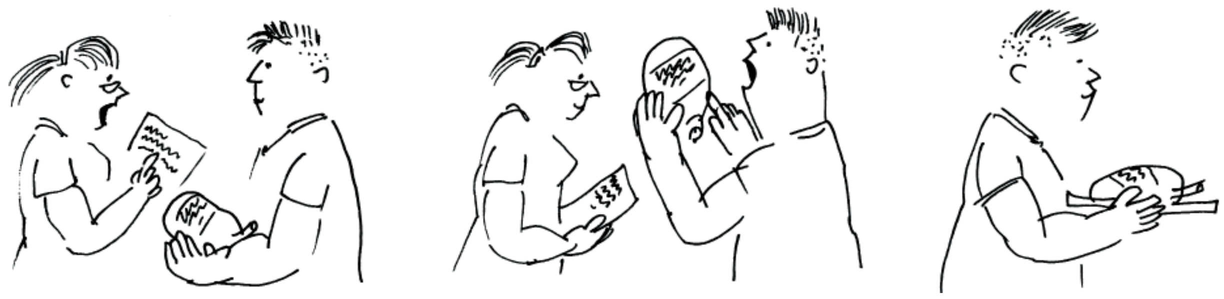

How appropriate is this type of DC to prevent medication errors	very appropriate // not appropriate at all
Is this type of DC performed at your unit?	yes routinely // only in exceptions // no (→ go to next scenario) // don't know
In how many of such are you personally involved in on an average working day, including cytostatics, potassium, antiemetics?	None // 1-5 // 6-10 // more than 10
How frequently do you detect errors, discrepancies, or inconsistencies during a double-check of this type?	daily or several times daily // weekly or several times weekly // monthly or several times monthly // few times per year // never)
If you were to decide for your unit: Would you eliminate this type of DC?	Yes // no keep as is // perform only in exceptions

© Stiftung Patientensicherheit Schweiz 2015

Scenario B Verification of order and IV bag:<br>Nurse A verifies data on IV bag label against the order (paper or computer screen). Later nurse B verifies data on IV bag label against the order (paper or computer screen) again. One of both administers the medication to the patient.	
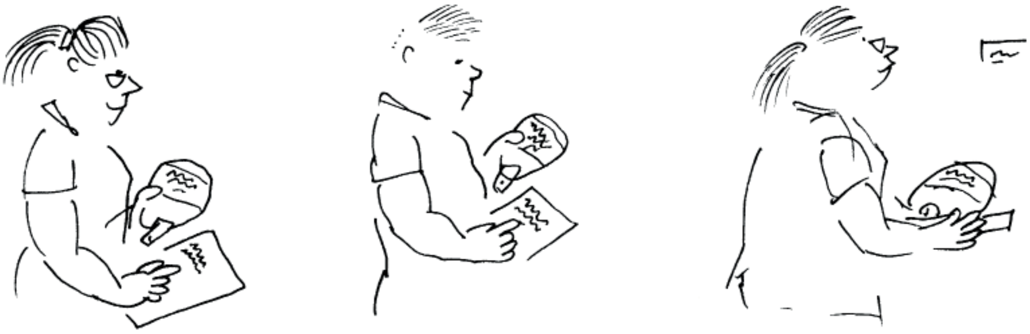	
How appropriate is this type of DC to prevent medication errors	very appropriate // not appropriate at all
Is this type of DC performed at your unit?	yes routinely // only in exceptions // no (→ go to next scenario) // don't know
In how many of such are you personally involved in on an average working day, including cytostatics, potassium, antiemetics?	None // 1-5 // 6-10 // more than 10
How frequently do you detect errors, discrepancies, or inconsistencies during a double-check of this type?	daily or several times daily // weekly or several times weekly // monthly or several times monthly // few times per year // never)
If you were to decide for your unit: Would you eliminate this type of DC?	Yes // no keep as is // perform only in exceptions

© Stiftung Patientensicherheit Schweiz 2015

Scenario C Local on-site preparation of infusions:<br>Based on the order, nurse A assembles drugs and solvents, calculates flow-rate and volume and writes down the results on labels. Nurse B verifies drugs and solvents against the order, repeats the calculations and confirms. One person prepares the infusions. Nurse A or B administers the medication to the patient.	
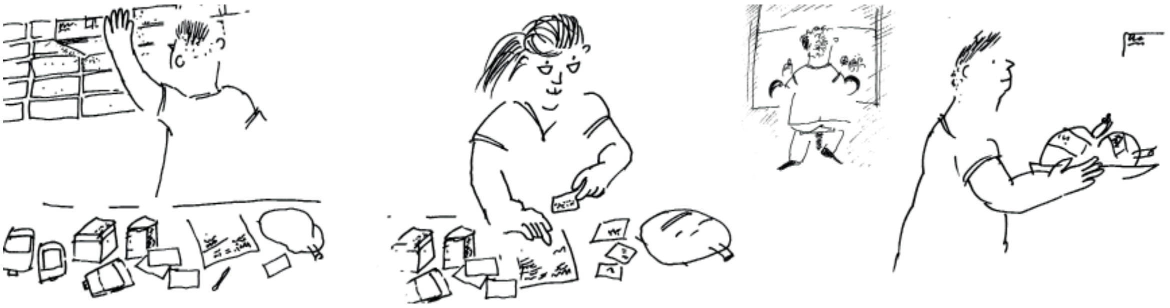	
How appropriate is this type of DC to prevent medication errors	very appropriate // not appropriate at all
Is this type of DC performed at your unit?	yes routinely // only in exceptions // no (→ go to next scenario) // don't know
In how many of such are you personally involved in on an average working day, including cytostatics, potassium, antiemetics?	None // 1-5 // 6-10 // more than 10
How frequently do you detect errors, discrepancies, or inconsistencies during a double-check of this type?	daily or several times daily // weekly or several times weekly // monthly or several times monthly // few times per year // never)
If you were to decide for your unit: Would you eliminate this type of DC?	Yes // no keep as is // perform only in exceptions
Does the second nurse already know the results of her colleague when she repeats the procedure (e.g., can she see the ticket with the number of tablets or the flow rate, etc.)?	Yes // no

© Stiftung Patientensicherheit Schweiz 2015

<table>
<tr><td colspan="2">Scenario D Calculation of flow-rate for infusions:
Nurse A calculates the flow-rate for the infusion and writes down the result. Nurse B repeats the calculation. She compares both results and confirms. One of both administers the medication to the patient.</td></tr>
<tr><td colspan="2">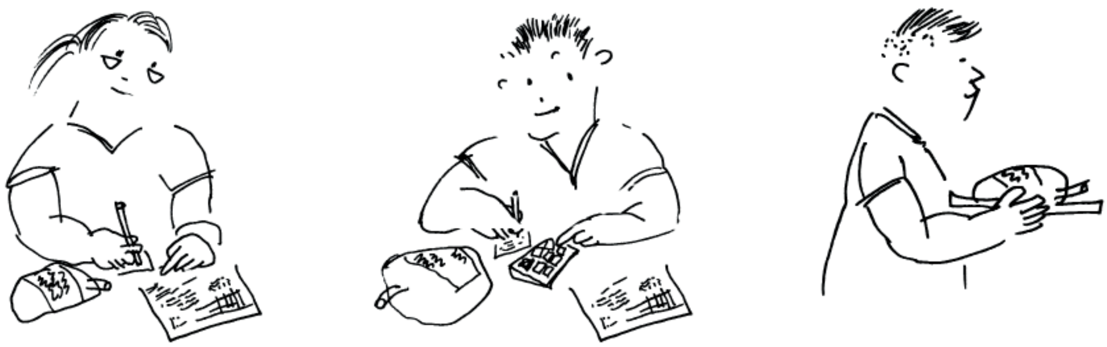</td></tr>
<tr><td>How appropriate is this type of DC to prevent medication errors</td><td>very appropriate // not appropriate at all</td></tr>
<tr><td>Is this type of DC performed at your unit?</td><td>yes routinely // only in exceptions // no (→ go to next scenario) // don’t know</td></tr>
<tr><td>In how many of such are you personally involved in on an average working day, including cytostatics, potassium, antiemetics?</td><td>None // 1-5 // 6-10 // more than 10</td></tr>
<tr><td>How frequently do you detect errors, discrepancies, or inconsistencies during a double-check of this type?</td><td>daily or several times daily // weekly or several times weekly // monthly or several times monthly // few times per year // never)</td></tr>
<tr><td>If you were to decide for your unit: Would you eliminate this type of DC?</td><td>Yes // no keep as is // perform only in exceptions</td></tr>
<tr><td>Does the second nurse already know the results of her colleague when she repeats the procedure (e.g., can she see the ticket with the number of tablets or the flow rate, etc.)?</td><td>Yes // no</td></tr>
</table>

© Stiftung Patientensicherheit Schweiz 2015

Scenario E Calculation and preparation of oral chemotherapies: Nurse A calculates the number of tablets against the order and prepares them. Nurse B repeats the calculation and verifies the prepared tablets. One of both dispenses the medication to the patient.	
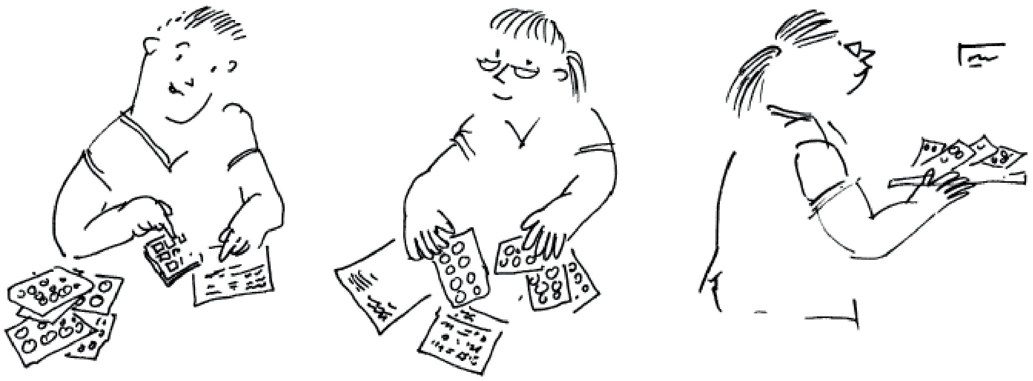	
How appropriate is this type of DC to prevent medication errors	very appropriate // not appropriate at all
Is this type of DC performed at your unit?	yes routinely // only in exceptions // no (→ go to next scenario) // don't know
In how many of such are you personally involved in on an average working day, including cytostatics, potassium, antiemetics?	None // 1-5 // 6-10 // more than 10
How frequently do you detect errors, discrepancies, or inconsistencies during a double-check of this type?	daily or several times daily // weekly or several times weekly // monthly or several times monthly // few times per year // never)
If you were to decide for your unit: Would you eliminate this type of DC?	Yes // no keep as is // perform only in exceptions
Does the second nurse already know the results of her colleague when she repeats the procedure (e.g., can she see the ticket with the number of tablets or the flow rate, etc.)?	Yes // no

© Stiftung Patientensicherheit Schweiz 2015

What constitutes the essential characteristic of a good double-check?	two persons check the medication together // two persons make the same checks successively // one person independently repeats a process (e.g., counting) without knowing the results of her preceding colleague
Please consider the number of the scheduled number of double-checks at your unit. Is the extent of double-checks good as is, or should there be more or less checks?	The extent of double-checks is good and appropriate as is // there should be additional double-checks // there should be fewer double-checks
Which of the following factors frequently interferes with performing a good DC? Choose all that applies	hurry and hectic at the unit // interruptions and distractions // noise and poor illumination in the medication room // problems to find a colleague for DC // overcrowded rooms // own fatigue
Are there internal guidelines explaining which checks are required for which medications at your unit?	Yes, and I know them well // Yes, but I do not know their contents well // No guidelines exist // I do not know
Are cytostatics prepared at your unit?	Yes // no
Are premedications double-checked at your unit?	not double-checked at all // routinely double-checked // only occasionally // don't know
If you were to decide for your unit: Would you opt in favor or against the routine double-checking of premedications?	Would opt in favor of double-checking of premedications // would opt against double-checking of premedications

Your thoughts about double-checking<br>*Please indicate your level of agreement* with the following statement.	completely agree // completely disagree
Nowadays, double-checking is regarded „good practice" in cancer care.	completely agree // completely disagree
The double-check introduces a final moment of silence and concentration before medications are being administered.	completely agree // completely disagree
A hospital needs to implement double-checking for dangerous drugs because otherwise it will look bad in case of error.	completely agree // completely disagree
Most medication incidents are attributable to a lack of attention of individuals.	completely agree // completely disagree
Most errors happen directly before medication administration, after all double-checks have been conducted.	completely agree // completely disagree
Technical solutions like barcode /bedside scanning would be better alternatives to double checks by humans.	completely agree // completely disagree
The double-check is the central task for medication safety.	completely agree // completely disagree
The resources needed for the double-check are justified for the additional safety it produces.	completely agree // completely disagree
Errors of single individuals can be intercepted with redundant checks by humans.	completely agree // completely disagree
The primary purpose of the double-check is to prevent errors in medication preparation.	completely agree // completely disagree
Responsibility is shared with the double-check.	completely agree // completely disagree
The double-check gives a false sense of safety.	completely agree // completely disagree
In everyday practice, the double-check is often superficial routine.	completely agree // completely disagree
I'm particularly vigilant if a double-check is required for a medication, because it must be a dangerous drug then.	completely agree // completely disagree

© Stiftung Patientensicherheit Schweiz 2015

Your thoughts about double-checking<br>*Please indicate your level of agreement* with the following statement.	completely agree // completely disagree
I'm convinced about the benefits of the double-check how it is done here.	completely agree // completely disagree
The primary purpose of the double-check is to detect prescribing errors.	completely agree // completely disagree
Environmental factors such as illumination, interruptions etc. increase the risk for errors considerably.	completely agree // completely disagree
If the double-check would be deimplemented, threats to patients would increase considerably.	completely agree // completely disagree

About your individual situation<br>*Please indicate your level of agreement* with the following statements	completely agree // completely disagree
I know what matters most for a good double-check.	completely agree // completely disagree
I can give my colleagues critical feedback when they are inattentive at the double-check.	completely agree // completely disagree
I'm sure that I make a good double-check despite pressure and interruptions.	completely agree // completely disagree
I know exactly which checks are required for a certain medication.	completely agree // completely disagree
It can happen to me that I forget the double-check.	completely agree // completely disagree
I'm certain that I do the double-check correct.	completely agree // completely disagree
I realize myself when I'm inattentive and 'asleep at the wheel'.	completely agree // completely disagree
To me, it feels reassuring to know that dangerous drugs which I prepared will be checked by a co-worker.	completely agree // completely disagree
I'm confident I detect every important inconsistency in medications.	completely agree // completely disagree
Sometimes I rely too much on the second person at the double-check.	completely agree // completely disagree
I often feel distracted from my work when I'm called to a double-check by a colleague.	completely agree // completely disagree

© Stiftung Patientensicherheit Schweiz 2015

Please indicate your level of agreement with the following statements	
There are very different views about the importance of double-checking at my unit.	completely agree // completely disagree
Staff at my unit is rather sceptical about double-checking.	completely agree // completely disagree
Rules at our unit are somewhat stricter that what we actually do in double-checking during routine care.	completely agree // completely disagree
There are times of the day or week where a good double-check is not feasible.	completely agree // completely disagree
We make too many checks of medications.	completely agree // completely disagree
The double-check makes our workflow more complicated.	completely agree // completely disagree
High quality of double-checking is very important to nursing leaders of my unit.	completely agree // completely disagree
High quality of double-checking is very important to physician leaders of my unit.	completely agree // completely disagree

Please indicate your level of agreement with the following statements	
It happens that two persons make the same mistake during a double-check (e.g., calculation error).	completely agree // completely disagree
Some colleagues here rely too much on the second person at the double-check.	completely agree // completely disagree
Misleading information or the way, orders are filled frequently complicate the double-check.	completely agree // completely disagree
The different speed of staff (e.g., in reading) makes the double-check difficult.	completely agree // completely disagree

At your unit….<br>*Please indicate your level of agreement* with the following statements	
How frequently is a scheduled double-check done only superficially, is not completed or not conducted at all?	several times daily // several times weekly // several times monthly // less frequently or never
How frequently does a patient get her medication from staff who was not involved in the DC of this medication?	several times daily // several times weekly // several times monthly // less frequently or never
How frequently are the medications of several patients double-checked in series without break?	several times daily // several times weekly // several times monthly // less frequently or never
How frequently are you interrupted in your own tasks to support a colleague doing a double-check?	More than 10 times per day // about 5 - 10 times per day // About 5 – 10 times per week less frequently or never
Were there one or more serious medication errors at your unit in the past 12 months?	Yes, several // yes, one // no, none // don't know
When you consider the last serious medication error: Could this error have been prevented with a good double-check?	Yes // likely // no /don't know

© Stiftung Patientensicherheit Schweiz 2015

Questions about you	
Are you …	Female // Male
How old are you?	….years
Qualification	Qualified nurse // Oncology nursing // expert // Head nurse // Other
Primary place of work	Ambulatory infusion unit // Oncology day care unit // Ward // Other
Years of practice in oncology	< 1 months // < 1 year // 1-5 years // 5-10 years // > 10 years
In a typical working week: How many hours do you work in direct patient care?	<10 hours / week // 10-25 hours week // 25-40 hours / week // > 40 hours / week
Do you personally have experience with barcode scanning (e.g., wristbands, blood products)	Yes / no

© Stiftung Patientensicherheit Schweiz 2015
